# Fourier Plane Tomographic Spectroscopy Reveals Orientation-Dependent Multipolar Plasmon Modes in Micrometer-Scale Janus Particles

**DOI:** 10.1021/acsnano.6c01771

**Published:** 2026-03-23

**Authors:** Felix H. Patzschke, Frank Cichos

**Affiliations:** Molecular Nanophotonics Group, Peter Debye Institute for Soft Matter Physics, 9180 Leipzig University, 04103 Leipzig, Germany

**Keywords:** Plasmonic Janus Particles, Fourier Optics, Spectroscopy, Surface Plasmons, Multipolar Resonances, Au, AuNP, BFP, LSP, LSPR, pJP, PS, SP

## Abstract

Precise control of light–matter interactions is a cornerstone of next-generation technologies, from ultrasensitive biosensing and single-molecule tracking to the development of adaptive metamaterials. While small, symmetric nanostructures are well-understood, micrometer-scale plasmonic Janus particles (pJPs), comprising dielectric cores with thin metallic caps, exhibit complex optical properties due to their asymmetric structure. Despite widespread applications in active matter research, their orientation-dependent scattering properties remain largely unexplored. We introduce Fourier plane tomographic spectroscopy for simultaneous four-dimensional characterization of scattering from individual micrometer-scale particles across wavelength, incident angle, and scattering angle. Combining measurements with finite-element simulations, we identify discrete spectral markers in visible and near-infrared regions that evolve predictably with cap orientation. Spherical-harmonics decomposition reveals that these markers arise from three distinct multipolar modes up to fifth order: axial-propagating transverse-electric, transverse-propagating transverse-electric, and transverse-propagating axial-electric, with retardation-induced splitting. We observe progressive red-shifts and line width narrowing of higher-order resonances, demonstrating curvature’s influence on mode dispersion. Orientation-specific scattering patterns exhibit polarization-dependent features enabling optical tracking of particle rotation. Beyond pJPs, this methodology establishes a general framework for characterizing asymmetric nanostructures of diverse material combinations and geometries, offering a toolkit for designing orientation-responsive nanoantennas, reconfigurable metasurfaces, active colloidal systems with tailored light–matter interactions, and high-precision optical tracking of particle rotation.

## Graphical abstract

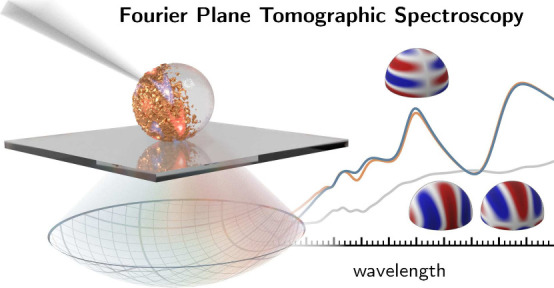

Precise control of light at the nanoscale through plasmonic excitations has emerged as a cornerstone technology enabling advances in next-generation sensing, energy harvesting, and quantum information systems. A significant aspect of these light–matter interactions is the excitation of surface plasmons (SPs)—collective oscillations of electrons at metal-dielectric interfaces.^1–3^ When these oscillations are confined to metallic nanostructures, they give rise to localized surface plasmons (LSPs) which generate highly localized enhancements of electromagnetic fields.^2–5^ These plasmonic phenomena find immediate application in critical challenges, from ultrasensitive biosensors for point-of-care medical diagnostics^6–8^ to efficiency-enhanced photovoltaic cells addressing global energy demands,^9–11^ and quantum plasmonic devices enabling imaging beyond the diffraction limit.^12–14^ The sensitivity of localized surface plasmon resonances (LSPRs) to geometry and local refractive index has been extensively studied in various nanostructures, including nanorods,^15,16^ nanotriangles,^17–20^ and nanoparticle aggregates.^21–24^ These nanostructures act as resonators, supporting a collection of fundamental oscillation modes. The resonance frequencies of these modes depend strongly on the size and shape of the underlying structure.^19,25–27^ Most research has focused on plasmonic structures smaller than or comparable to the probing wavelengths, which allows for straightforward excitation and analysis of fundamental LSPRs.^4,13,28^

Meanwhile, higher-order resonances, which provide valuable information about the spatial characteristics of plasmonic oscillation modes, have rarely been studied. While they are known to appear as contributions to plasmonic excitations on various nanostructures,^29,30^ investigating these higher-order LSPRs in the regime where they constitute principal features, requires larger plasmonic structures, where higher order modes of the fundamental LSP modes can couple to incident visible light fields.^16,31–33^ Structures with reduced symmetry are particularly interesting, as they split fundamental LSP modes along their principal axes^34,35^ and exhibit multipolar resonances that map to geometric features.^36^ Analysis of the anisotropic optical response of such nanostructures offers unique insights into orientation-dependent light–matter interactions.^20,35–39^ Micrometer-sized plasmonic Janus particles (pJPs) are ideal candidates for studying fundamental LSPR modes in the optical regime.^40^ These particles, consisting of a dielectric core with a thin metallic coating on one hemisphere, bear strong structural resemblance to spherical cap systems, enabling efficient SP coupling^41^ and exhibit multiple LSP modes with orientation-dependent excitations.^34,42^ While classical Mie theory^43^ only makes predictions for maximally symmetric particles, it provides a valuable reference framework for the interpretation of findings regarding higher-order plasmonic modes of fundamental LSPRs. The strong plasmonic responses of pJPs have enabled their widespread use in studying self-organization and active matter.^44,45^ Theoretical and numerical studies have revealed complex light–matter interactions in these systems, predicting counterintuitive phenomena such as stable rotation induced by linearly polarized light fields.^46^

Despite their widespread use in active matter research, the orientation-dependent plasmonic responses of individual micrometer-scale pJPs remain incompletely characterized, limiting their potential for precision optical manipulation and sensing applications. Here, we address this critical knowledge gap by developing Fourier plane tomographic spectroscopy, building on back focal plane (BFP) principles,^33,47,48^ to provide comprehensive four-dimensional characterization of individual pJP light–matter interactions. We investigate pJPs and map the intensity of scattered light resolved for wavelength and scattering angle while varying the direction of illumination. This approach reveals the contributions of higher-order excitations of specific SP modes to the optical responses. Complementing our experimental work, we perform numerical simulations that show excellent agreement with measurements, allowing us to correlate peaks in the scattering spectra to orientation-dependent surface plasmon modes.

This quantitative understanding of orientation-dependent plasmonic responses provides essential design principles for emerging applications in active metamaterials, optical manipulation systems, and next-generation sensing platforms where precise control over light–matter interactions is paramount. Through this combined approach, we gain new insights into the light–matter interactions of large, anisotropic, plasmonic structures in an intermediate regime between two well-understood limits—the dipole resonances of small particles, where incident optical fields are effectively homogeneous,^49,50^ and the broadband SP excitations on extended thin films.^51^ This comprehensive framework enables rational design of orientation-responsive plasmonic devices and provides a pathway toward next-generation metamaterials with dynamically tunable optical properties.

## Results

We investigated the light–matter interactions of pJPs using a spectroscopic technique developed for this purpose: Fourier Plane Tomographic Spectroscopy. This method builds upon established BFP spectroscopy techniques, which exploit the optical Fourier transform performed by lenses between their front and back focal planes.^52^ Therein, each lateral wave vector component (emission angle) in the sample plane maps to a specific position in the BFP and *vice versa*: Light scattered at an angle θ relative to the optical axis appears at a radial coordinate *r* = *n*·*f*·sin θ in the BFP, where *f* is the focal length and *n* the refractive index of the immersion medium.^47,48^ Conversely, the same Fourier relationship applies to the illumination pathway, enabling precise control of incident light direction with an aperture in the condenser BFP.^33,53^ Crucially, selecting a specific incidence direction establishes the reference axis for measuring scattering angles.

By spectrally dispersing the angular information in the BFP, this method simultaneously resolves wavelength and scattering direction. This approach provides comprehensive four-dimensional characterization of asymmetric particles’ optical response, capturing the full angular and spectral distribution of scattered light under controlled illumination conditions.

### Fourier Plane Tomographic Spectroscopy

Angle-resolved scattering spectra were acquired utilizing a custom-designed optical configuration, depicted in Figure 1A, constructed around a standard dark-field microscope. In the illumination pathway, a precision aperture (B1), in conjunction with the dark-field condenser (B2), constrained the incident illumination to within 0.096 sr solid angle along predetermined directions. The instrument’s optical path was configurable to either directly image the sample plane or project the back focal plane (B3) of the objective lens onto an intermediate imaging plane. A spatial filter (B4) positioned at an intermediate image plane isolated scattered light exclusively from the particle under investigation, and spectral dispersion was accomplished via a transmission grating. Full details of the optical configuration and sample preparation are provided in the Methods section; measurement procedures are expanded upon in Section S1 of the Supporting Information.

**Figure 1:**
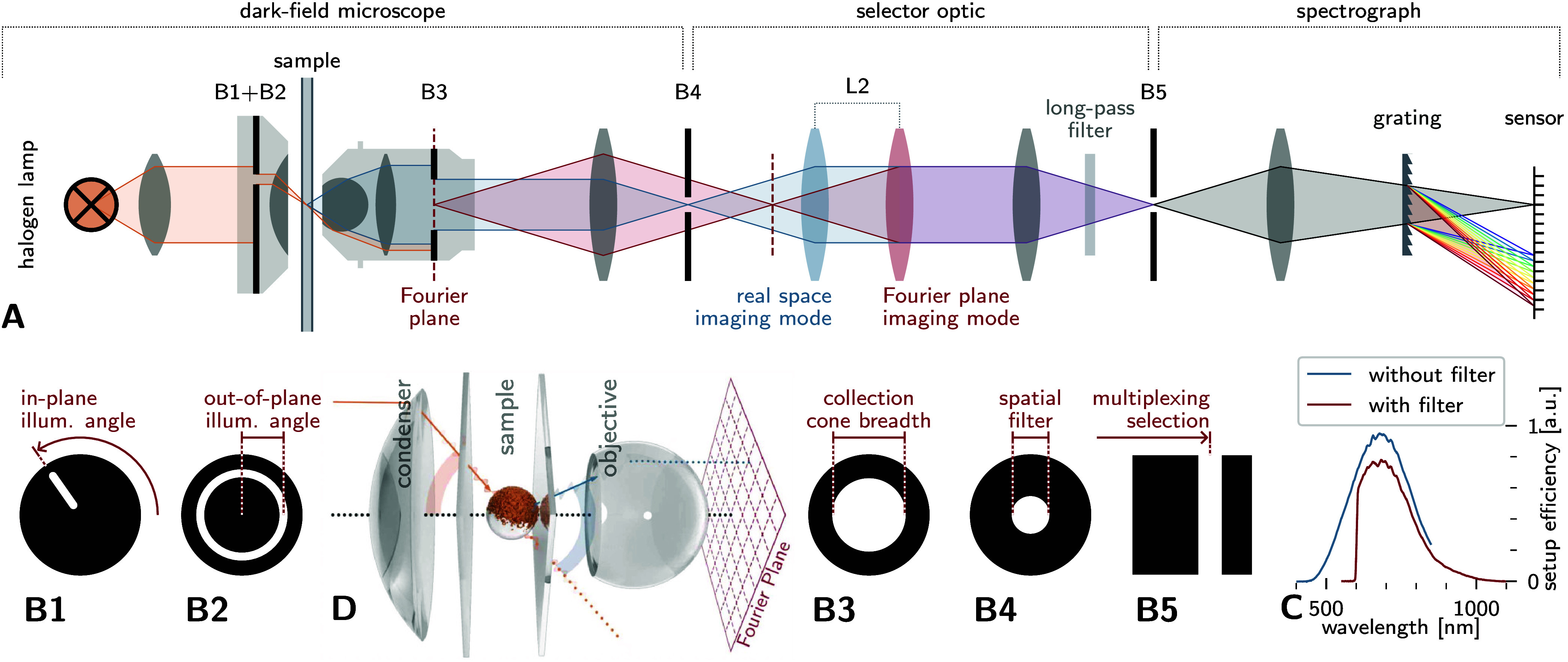
**A:** Schematic of the imaging light path. **B1–B5:** Schematics of the apertures. B1–B3 lay in Fourier planes, B4 lies in an image plane. Depending on the placement of the lens L2, either the image plane or the Fourier plane may be imaged onto B5, and subsequently the camera sensor. **C:** Spectral response function of the setup. Measurements with and without the long-pass filter were spliced together to obtain spectra covering the entire sensitivity range. **D:** Immersion oil was used both as interfacing medium between samples and optics, and as the ambient medium inside the samples. The oil matches the refractive index of the glass coverslips, ensuring a nearly homogeneous optical environment (Δ*n* ≤ 0.013) around particles under observation.

Real-space imaging facilitated the selection of individual particles and spectral measurements without angular resolution, while back focal plane (BFP) imaging provided spectrally resolved Fourier-space scattering distributions. To acquire these data sets, the slit B5 was systematically translated across the BFP image during the recording process, enabling the camera to sequentially capture vertical lines of spectrally dispersed scattering information.

For spectral calibration, the objective’s back aperture (B3) was fully opened to its maximum numerical aperture of 1.3, allowing direct transmission of light from the dark-field illumination pathway into the imaging system. The resulting spectral efficiency curves of the optical configuration are presented in Figure 1C. The spectral range of the measurements was constrained at shorter wavelengths by the emission spectrum of the light source, while the upper wavelength limit was determined by the quantum efficiency of the sCMOS detector and partial absorption of near-infrared radiation by the optical components in the beam path.

Samples comprised immobilized plasmonic particles sandwiched between two glass cover slides (*n* = 1.519 ± 0.012) and embedded in immersion oil (*n* = 1.518). This configuration, as illustrated in Figure 1D, minimizes refractive index discontinuities, providing an effectively homogeneous environment (Δ*n* ≤ 0.013) around the particles, suppressing perturbations through boundary effects such as thin-film interference.

### Single Gold Nanoparticle Spectra

To validate the spectroscopic performance of our experimental setup, we conducted measurements on gold nanoparticles (AuNPs) with a diameter of 65 nm. Figure 2A demonstrates the excellent agreement between our measured scattering spectra and theoretical predictions based on Mie theory,^43^ utilizing the complex refractive index of gold reported by Johnson and Christy.^54^ Statistical analysis of the measurements revealed minor variations in peak positions (2.2 nm standard deviation) attributable to the particle size distribution, alongside a systematic red-shift of 3.6 nm relative to theoretical predictions. For particles substantially smaller than the incident wavelength (characterized by a size parameter *x* = (2*πnr*)/λ ≪ 1), the dipole approximation predicts angular scattering distributions that are invariant with respect to shape, resulting in measured intensities that directly correspond to total scattering cross sections. In our experimental configuration, the size parameter ranges from 0.34 at λ = 900 nm to 0.77 at λ = 400 nm, which deviates somewhat from the strict dipole approximation regime. This deviation explains the observed red-shift of the measured resonance peak: multipole contributions to the angular distribution of scattered light become progressively less significant at longer wavelengths, resulting in proportionally greater collection efficiency through the objective back aperture in this spectral region.

**Figure 2:**
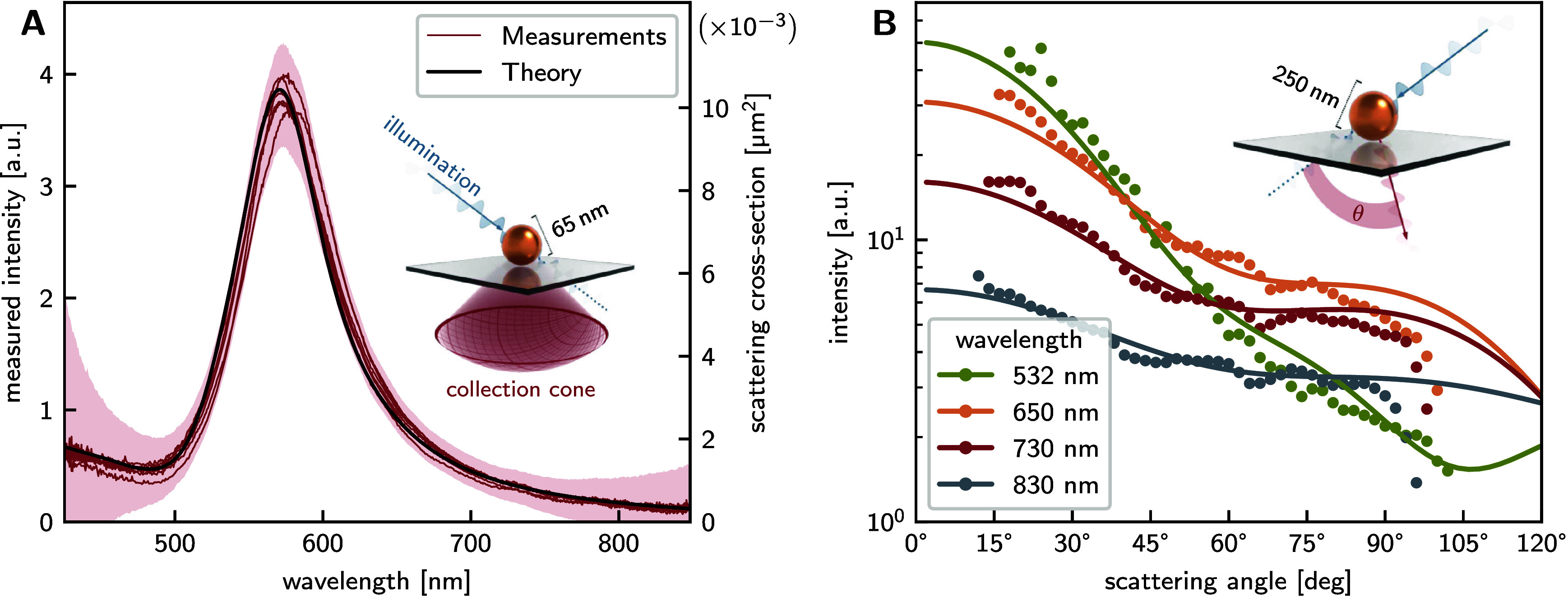
Validation measurements. **A:** In an angle-accumulated measurement, a cone of scattered light is collected (see inset) and spectrally analyzed at once. For a 65 nm AuNP, the theoretical scattering cross-section is well-approximated by this, falling within a standard deviation (shaded area) of the measurements. **B:** In an angle-resolved measurement (see inset), the light emitted under a specific scattering angle θ is isolated before spectral dispersion. For a spherical AuNP (*d* = 250 nm), the measured angular intensity profiles (points in diagram) match the predictions of Mie theory^43^ (lines in diagram) for various wavelengths. All measured intensities were scaled by the same constant factor to match the theory curves. This demonstrates the accurate capture of the system’s response in both the θ- and the λ-dimension.

For angle-resolved measurements, we used larger AuNPs, 250 nm in diameter. Particles in this size regime exhibit wavelength-dependent multipolar scattering, producing nondipolar angular structure and enabling validation of angular resolution. The measured angular scattering patterns are in strong agreement with predictions of Mie theory,^50,55^ as we demonstrate in Figure 2B. As the wavelength decreases, the size parameter increases from *x* = 1.2 at λ = 1000 nm to *x* = 3.0 at λ = 400 nm, leading to increasingly forward-directed scattering.

In contrast to integrated measurements, angle-resolved detection distributes the scattered photons over many angular bins on the camera sensor, substantially reducing the number of detected photons per bin. The maximum usable exposure per frame is constrained by sensor saturation in the brightest spectral and angular regions. 250 nm AuNPs were selected to ensure sufficient photon counts across all bins. Though larger than the particles used for spectroscopic validation, they are still significantly smaller than the pJPs studied subsequently. The solid agreement between measured and theoretical angular patterns demonstrates that the practical lower size limit imposed by photon statistics and detector dynamic range (see Supporting Information, Section S2) lies well below the particle sizes investigated in this work.

### Illumination-Angle-Dependent Scattering Intensity

Micron-sized pJPs exhibit remarkably complex angle-dependent scattering behavior arising from their structural anisotropy, despite maintaining a spherically symmetric core. Our experimental configuration enables precise manipulation of illumination angles through controlled rotation of aperture B1 within the illumination pathway while preserving fixed particle orientation throughout measurements.

Prior to inserting aperture B1, we recorded a standard dark-field image of a single pJP (Figure 3A). This image allows us to identify the symmetry plane which we then used to define the in-plane illumination angle for all subsequent measurements. Figure 3B–M present dark-field images of the same pJP under 12 different illumination angles, demonstrating how the light-scattering activity is localized based on the orientation of the pJP and the direction of illumination. When light is incident from the Au side (Figure 3B–F), the scattered intensity is strongly localized, characteristic of plasmonic scattering from the metallic surface. As the pJP is illuminated side-on (Figures 3G–H), the arc of the cap exhibits more even brightness, scattered light intensity being less localized. The PS side exhibits minimal scattering due to its low refractive index contrast with the surrounding medium. Under PS-side illumination (Figure 3I–M), scattering is observed from both hemispheres, separated by a distinctive dark region. This pattern can be attributed to plasmon-mediated scattering from the Au cap coupled with subsequent refraction through the PS core.

**Figure 3:**
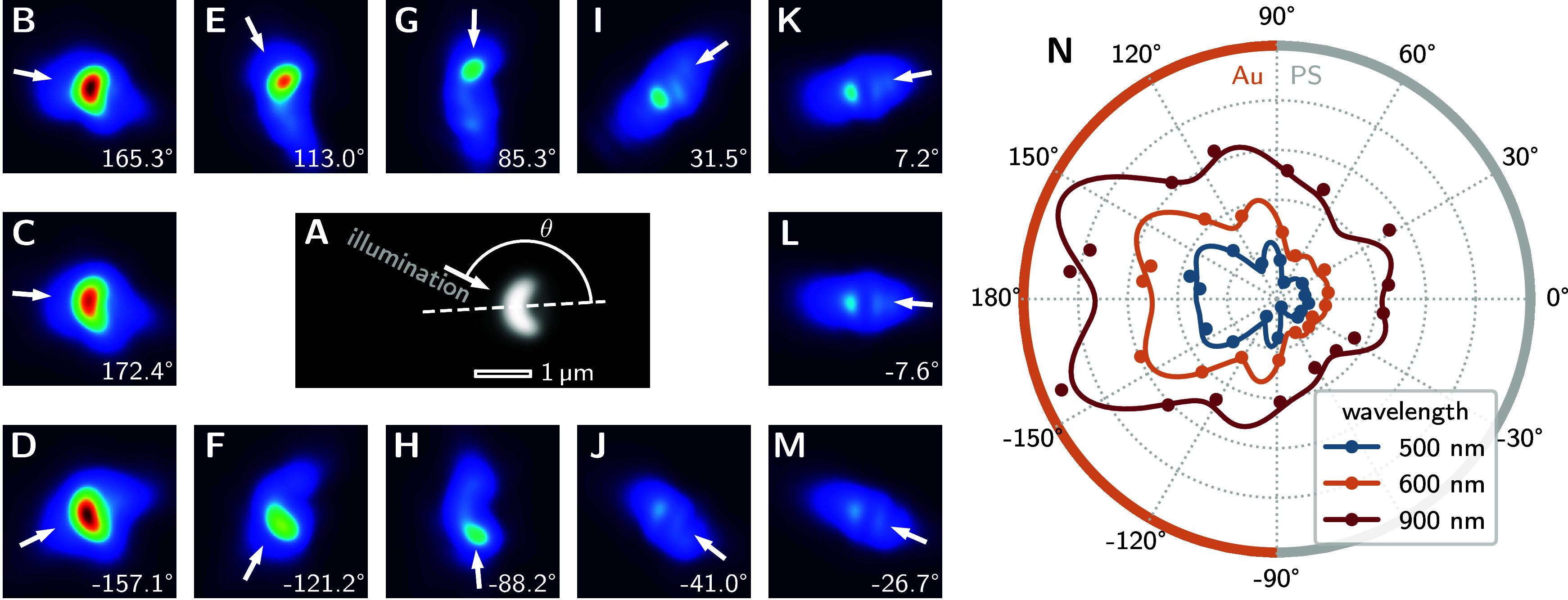
**A:** A standard dark-field image of a pJP. The in-plane illumination angle (here θ) is defined for subsequent measurements w.r.t. the symmetry plane as indicated by the dashed line. **B–M:** Dark-field images of the same pJP under various selective illumination modes. The respective in-plane illumination angles are indicated by arrows and noted in the lower right corner. **N:** Apparent brightness of the pJP depending on the in-plane illumination angle, for different wavelengths. The profile lines were obtained by fitting to a finite Fourier series. The colored rim indicates which side of the JP the light is incident on for a given angle.

The apparent brightness of the pJP in dark-field imaging depends systematically on both illumination direction and wavelength. Analysis of spectrally resolved scattering under restricted illumination conditions reveals wavelength-dependent angular profiles (Figure 3N). The scattering intensity is consistently higher for Au-side illumination across all wavelengths, with the intensity contrast between Au-side and PS-side illumination being most pronounced at shorter wavelengths. The angular profiles at shorter wavelengths display multiple well-defined extrema, while these features become less distinct at longer wavelengths, consistent with the transition between different scattering regimes.

### Single pJP Scattering Spectra

The recorded scattering spectra of individual pJPs under selective illumination from specific directions (Au side, PS side, and perpendicular to the symmetry axis) are shown in Figure 4A. In the wavelength range from 600 to 1000 nm, the scattering intensity increases monotonically for all illumination geometries. The measured scattering response was consistently highest for illumination from the Au side, as evidenced by the angular profiles. The Au-side illumination spectrum exhibits a distinct spectral shoulder at 550 nm. This feature appears diminished under perpendicular illumination and is absent when illuminating from the PS side.

**Figure 4:**
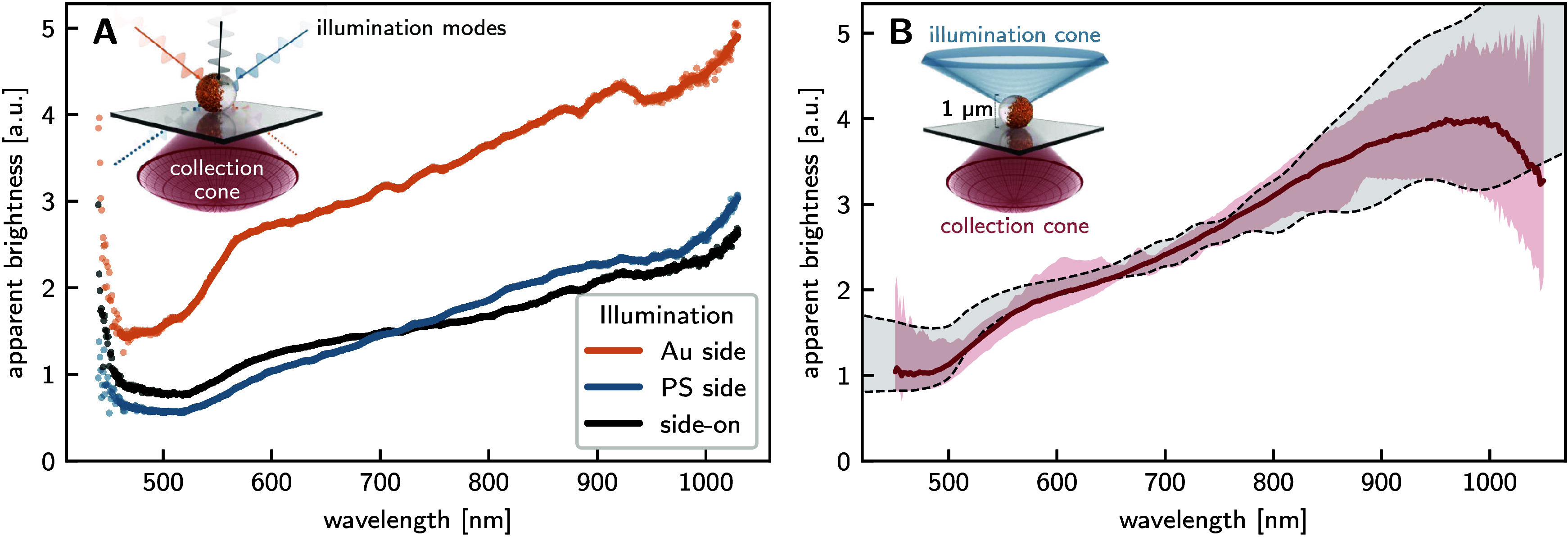
Measured scattering spectra of 1 μm pJPs. **A:** Scattering spectra of a single pJP, with different settings of the selective illumination. Light was incident from the Au side (yellow curve), from the PS side (blue curve) and side-on (black curve). **B:** Under standard dark-field illumination, light is incident from a range of directions simultaneously. The red line depicts the mean of the measured dark-field spectra of individual pJPs with the shaded area corresponding to the range of all measurements. The black dashed lines bound the range of simulated dark-field spectra.

Figure 4B illustrates the collective dark-field scattering spectra acquired from multiple pJPs juxtaposed with corresponding finite-element simulations that incorporate the objective’s numerical aperture constraints. Quantitative comparison reveals excellent correlation between experimental measurements and computational predictions, with the experimentally obtained spectral profiles consistently falling within one standard deviation of the mean simulated response throughout the predominant wavelength domain examined. This robust agreement validates our numerical approach and confirms the underlying physical mechanisms governing the plasmonic response of these asymmetric structures.

Both measured and simulated spectra exhibit a consistent monotonic increase in scattering intensity across the 500–1000 nm spectral range, with a characteristic shoulder feature at 550 nm evident throughout all experimental observations, although its significance and position vary, as we show in Section S3 of the Supporting Information.

At wavelengths exceeding 900 nm, the measured spectra exhibit decreased signal-to-noise ratio. This spectral artifact stems predominantly from the inherent detector noise limitations, as the quantum efficiency of our detection system diminishes significantly in this near-infrared region. Notably, while our experimental measurements consistently demonstrate decreasing scattering intensity approaching negligible levels at longer wavelengths, computational simulations predict continued enhancement in scattering efficiency beyond this spectral boundary. This discrepancy between experimental observations and theoretical predictions warrants further investigation into near-infrared plasmonic behavior.

### Angular Scattering Intensity of pJPs

Fourier Plane Tomographic Spectroscopy provided comprehensive characterization of the angular distribution of scattered light intensity. We conducted measurements under three precise illumination configurations: Au-side axial, PS-side axial, and side-on illumination (illustrated in Figure 5 insets). For rigorous quantitative comparison between experimental measurements and computational simulations, we extracted one-dimensional intensity profiles along the polar coordinate from the Fourier plane images. Figure 5 presents both the measured and simulated scattering profiles.

**Figure 5:**
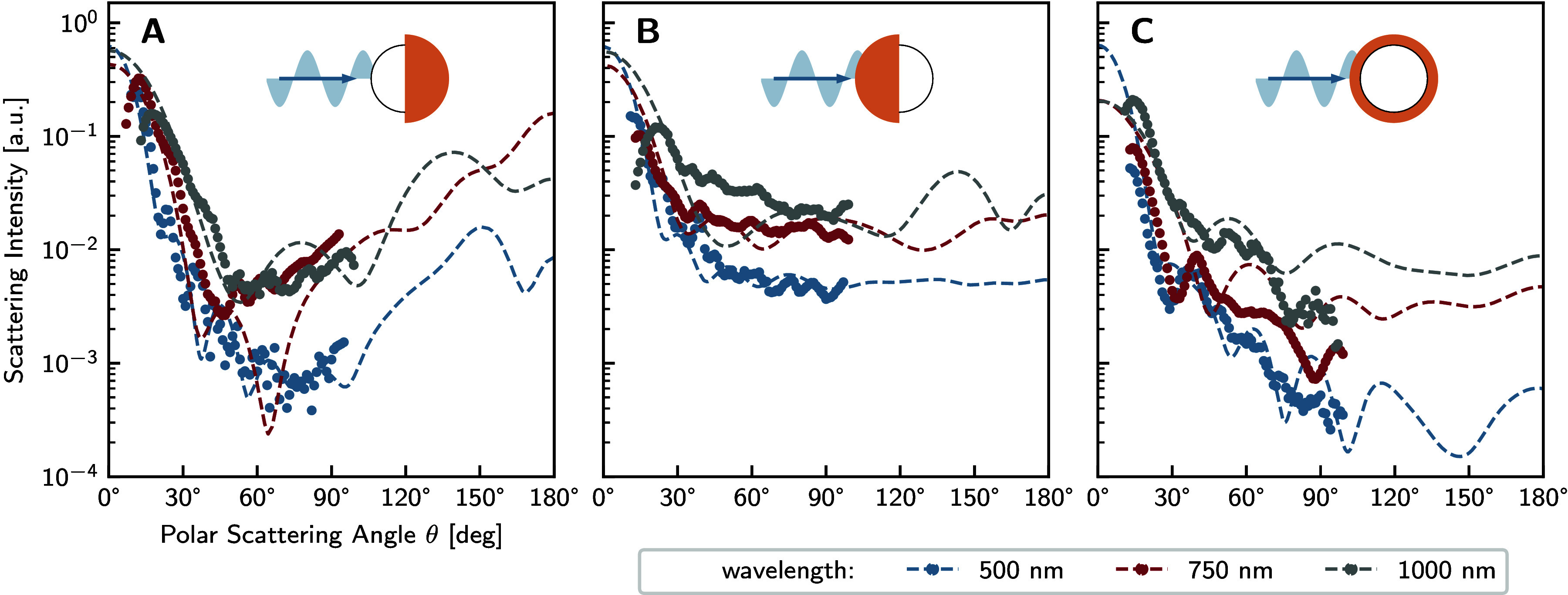
Scattering intensity of the pJP versus scattering angle for various wavelengths. The points correspond to measured intensities while the lines are simulation results. **A:** PS-side illumination. **B:** Au-side illumination. **C:** side-on illumination.

To assess agreement between simulation and experiment, we calculated the normalized root-mean-square deviations of the angular profiles. We found values to range between 1.06% and 9.45%, indicating solid agreement. As laid out in Section S4 of the Supporting Information, the visible discrepancies occur primarily where simulations predict particularly low intensities. There, the signal may be obscured by noise caused by imperfections of the real pJPs cap.

Consistent with established Mie scattering principles, the scattered light intensity demonstrates pronounced forward (θ = 0) directionality across all illumination geometries. However, the angular distributions exhibit distinct qualitative features that remain consistent throughout the analyzed spectral range. Under PS-side illumination (Figure 5A), the forward scattering peak achieves maximum intensity, followed by a characteristic minimum between 45° and 60° scattering angle, with subsequently increasing intensity at larger angles. For Au-side illumination (Figure 5B), the forward scattering peak exhibits significant broadening, and notably, the intensity stabilizes at a plateau value for intermediate scattering angles rather than continuously decreasing. This broader angular distribution yields higher total scattered intensity when integrated across all angles. Under transverse illumination (Figure 5C), the scattered intensity demonstrates a steep, nearly monotonic decrease with increasing scattering angle. These characteristic features are accurately reproduced in the simulated far-field patterns, providing robust validation of our experimental observations.

Side-on illumination breaks the geometric parity of the system, so that the light–matter interaction also exhibits nontrivial behavior w.r.t. the azimuthal scattering angle. Corresponding intensity profiles are discussed in Section S5 of the Supporting Information. In both simulation and experiment, we observe that scattered light is preferentially emitted in the direction of the Au cap, rather than that of the PS side, in this case.

## Discussion

### Scattering Spectra and Comparison to Mie Theory

To complement our experimental findings, we performed finite-element simulations to calculate the far-field scattering intensity patterns of pJPs under different illumination directions. In Figure 6A, we demonstrate that the measured scattering spectra can be reproduced well, using the simulation results. Variations between measured dark-field spectra were expected due to the unknown out-of-plane orientation of the pJP and NA of the objective, which had to be manually opened and closed again between measurements. These uncertainties were taken account of in the simulations: We generated a family of synthetic dark-field spectra, each simulated for different combinations of the unknown parameters.

**Figure 6:**
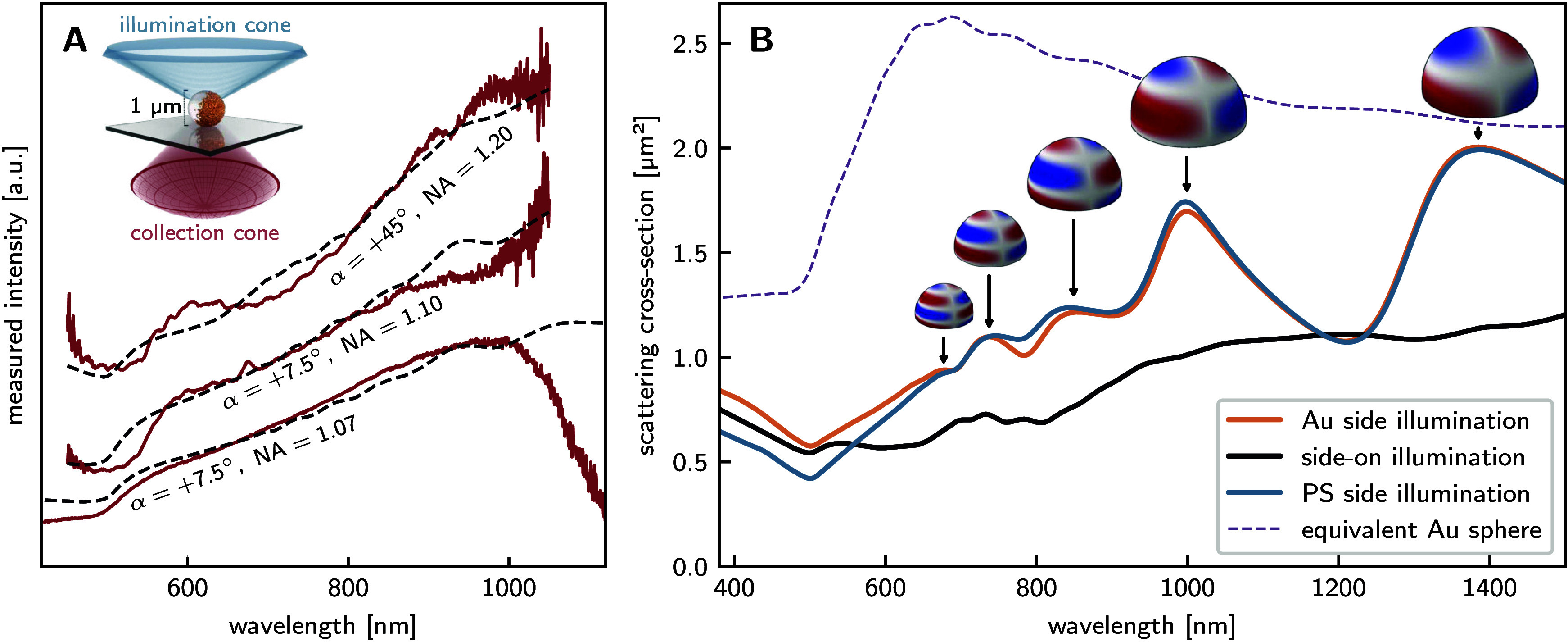
**A:** A selection of measured dark-field spectra of pJPs. Variations between spectra are due to the NA setting of the objective not being precisely known, as well as the out-of-plane orientation of different pJPs. Each measured spectrum (solid red lines) is paired with the closest-matching simulated spectrum (dashed black lines), chosen from a family of spectra simulated with varied NA and out-of-plane angle to account for the stated uncertainty. Positive out-of-plane orientations (here α) correspond to the gold cap pointing partially upward. **B:** Simulated scattering spectra of the pJP under illumination from the Au side (yellow), from the PS side (blue) and side-on (black). The dashed line indicates the scattering spectrum of an equivalently sized Au particle. Its less pronounced and more tightly spaced peaks resemble those of the pJP’s scattering spectrum under side-on illumination. Peaks in the spectra for axial illumination are labeled with sketches of the associated surface plasmons, the red and blue shaded areas indicating regions with opposite surface charge densities on the surface of the cap.

To that end, the finite numerical aperture of the real optics and the range of illumination angles in the dark-field configuration had to be taken into account. In contrast, the scattering spectra for unidirectional illumination modes and accumulation over all scattering angles are presented in Figure 6B. Although the simulated spectra show some notable differences from the measured dark-field spectra, key features are preserved between both data sets. The general increasing trend in scattering intensity with wavelength is maintained, including the characteristic upturn at 500 nm. These spectral profile differ markedly from homogeneous Au spheres of equivalent dimensions, which characteristically display a pronounced resonance peak followed by a slowly decaying intensity at longer wavelengths.^43,50,54^

For both Au-side and PS-side illumination, the simulated scattering cross sections exhibit wavelength-dependent behavior similar to the measured dark-field spectra, with a minimum at 500 nm followed by monotonic growth at longer wavelengths. However, the simulated spectra reveal distinct resonance peaks that are not readily apparent in the experimental measurements. Under side-on illumination, the simulated scattering cross-section increases more gradually with wavelength, showing less pronounced spectral features except for a cluster of minor peaks between 700 and 800 nm. The presence of less pronounced peaks in closer proximity to each other under this illumination mode indicates the excitation of a more complex LSP mode structure. In the interest of completeness, corresponding extinction spectra are provided as Supporting Information (Section S6). Dominated by the scattering contribution, they show highly similar spectral features.

The choice theoretical framework for the description of light–matter interactions with a size parameter close to 1 is the theory of Mie, which presents an analytical solution of the electromagnetic wave equation for an incident plane wave and a homogeneous, spherical particle.^43^ However, the lower degree of symmetry of our pJP w.r.t. a similar-sized gold sphere, and coupling between the outer and inner surfaces of the pJP’s cap effect a significantly altered plasmonic activity and, consequently, scattering response.

In the scattering spectrum of a solid Au sphere of equivalent size to the pJP we considered, the distinct peaks which we find for axial illumination, are not discernible, as we show in Figure 6B. This is due to the higher degree of symmetry of the sphere: Where the principal LSP modes are split by orientation for the pJP,^34^ on a sphere, all modes are 3-fold degenerate and can be excited by light incident from any direction. Consequently, the resonances of many different LSP modes overlap in the scattering spectrum of the gold sphere, as they do in the side-on-illuminated scattering spectra of the pJP.

The angular intensity distributions exhibit the same qualitative behavior for the pJP as those that one might calculate for a solid Au sphere: As the wavelength increases, the nonglobal maxima beside the consistently present forward-scattering peak become more well-distinguished in magnitude and fewer in number. The same happens as the direction of illumination is changed from side-on to axial. Both parameter changes can, in the context of Mie theory, be understood as a decreasing size parameter and thus the transition away from the ray optics regime (λ ≪ *R*) and closer to the scattering dipole model (λ ≫ *R*).

### Surface Plasmon Modes of the Gold Cap

The scattering properties of metallic nanostructures arise from the excitation of localized surface plasmons on their surfaces.^56^ Previous studies on smaller structures of similar geometry have shown that in the long-wavelength limit where *k* ≪ *R*_JP_^–1^, the response is dominated by two dipole modes: transverse-electric (TE) and axial-electric (AE). The TE mode is twice degenerate due to the rotational symmetry of the cap about its axis.^34^

Here, the larger size of the particle enables the excitation of higher-order LSP modes on the gold cap: The signature dipolar plasmon resonance typically prominent in smaller AuNPs manifests merely as a subtle shoulder in our spectral data, subsumed by the dominant trend of increasing scattering intensity with wavelength. In contrast to standard electromagnetic multipole decompositions that classify modes by electric and magnetic multipole order, we employ a geometry-adapted spherical-harmonic description of electric surface charge oscillations that is specific to the asymmetric cap geometry considered here, by developing a comprehensive mapping of the hemispherical cap geometry to the unit sphere $S^{2}$ . This mapping is constructed by projecting the outer (gold-oil interface) and inner (gold-polystyrene interface) surfaces of the cap onto the complete unit sphere $S^{2}$ , with the outer surface corresponding to the upper hemisphere ([0, π/2] ∋ θ → θ) and the inner surface to the lower hemisphere ([π/2, π] ∋ θ → π – θ ∈ [0, π/2]). Therein, the boundaries of either subdomain at θ = π/2, representing the physical rim where the gold film terminates, are identified.

This spherical mapping shows constructively, that the domain of the LSP modes is topologically equivalent to the unit sphere and thus enables us to express the fundamental spatial oscillations of the surface charge density in terms of spherical harmonics $Y_{l}^{m}\left(\theta ,\phi \right)$ , where θ and ϕ are spherical coordinates parametrizing $S^{2}$ . For resonant surface plasmon modes, this topology leads to a quantization of allowed wave numbers, approximated by

$$k_{\mathrm{SP}}=\frac{l}{R_{\mathrm{JP}}}$$

where *R*_JP_ is the effective radius of the gold cap. This relation describes standing wave resonances on the cap surface.

While the multipole *order* l determines the total spatial frequency of the oscillation, *m* represents the number of complete oscillations of $Y_{l}^{m}\left(\theta ,\phi \right)$ along a closed azimuthal curve at fixed θ, as evident from the analytical form of the spherical harmonics.^57^ Along a meridional path (fixed ϕ), the number of oscillations is given by l−|m| . Therefore, the pair (l−|m|,m) can be interpreted as the components of the surface plasmon wave vector in polar and azimuthal directions, respectively.

Though the cap’s surface is topologically equivalent to that of a spherical particle, its hemispherical geometry and broken inversion symmetry split the allowed oscillations into three distinct surface plasmon modes. Characterized by the direction of charge density oscillation and the direction of plasmon propagation, they cannot be uniquely classified by multipole order alone. The oscillation direction is determined by the polarization of the incident electric field, while the propagation direction is set by its wave vector. We therefore identify:

i) the axially propagating, transverse-electric (APTE) mode (Figure 7A, left column): This mode is excited when the electric field is polarized perpendicular to the particle’s symmetry axis. Due to cylindrical symmetry around this axis, the surface charge distribution must transform as a dipole under rotation—maintaining one complete oscillation around any circle of fixed θ. This requirement arises from the transverse dipolar nature of the excitation field coupling to the surface plasmon, which necessitates *m* = ± 1 as the only possible values that preserve the dipolar field symmetry while allowing propagation along the axis. This mode is doubly degenerate due to rotational symmetry about the particle axis.

**Figure 7:**
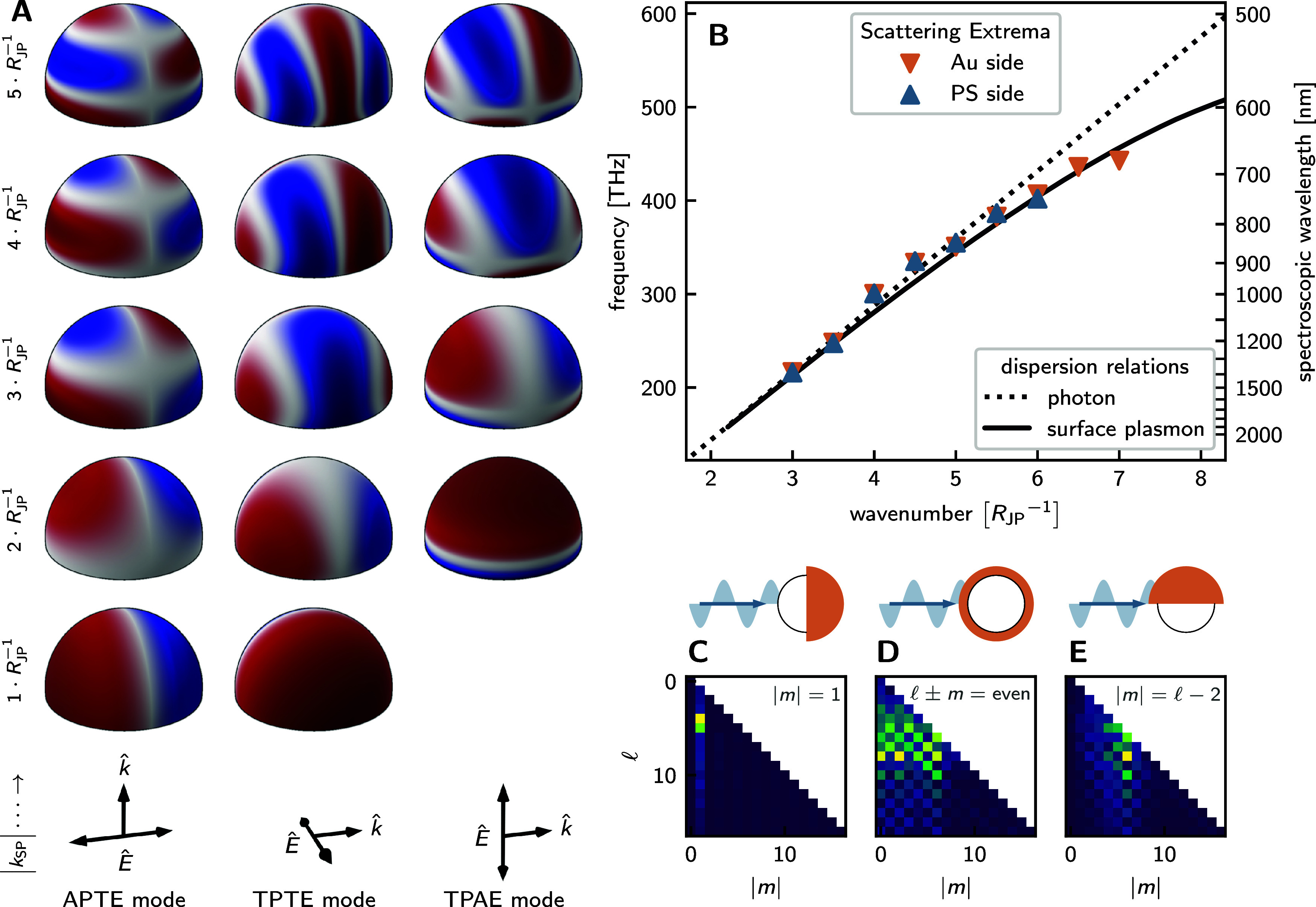
**A:** Sketches of the surface plasmon modes of a hemispherical gold cap. The red and blue shaded regions signify domains of pairwise opposite surface charge densities. The given wavenumber corresponds to the fundamental standing wave. The arrows at the bottom indicate the orientation of the outside light field. The bottommost modes of each column are those that would be excited in a spatially invariant external electric field. **B:** Excitation wavelengths of peaks and valleys in the axial illumination scattering spectra vs spatial frequency of the electric field on the surface of the Au cap. The inferred wavenumber is given in units of inverse pJP radii, 505 nm. **C–E:** Decompositions of the electric field on the cap’s surface for an excitation wavelength of 892 nm into spherical harmonics. The heatmaps visualize the amplitudes of each component $Y_{l}^{m}$ of the expansions. In **C**, the orientation promotes APTE mode excitation. The nonzero contributions fulfill |*m*| = 1. **D** and **E** correspond to the TPTE and TPAE modes, with the significant contributions matching the selection rules l±m=even and |m|=l−2 , respectively.

ii) the transverse-propagating, transverse-electric (TPTE) mode (Figure 7A middle): For the TPTE mode, the surface charge density must have odd symmetry about the plane orthogonal to the polarization direction. The coupling between the inner and outer hemispheres requires that, on the entire unit sphere, there must be an even number of closed azimuthal curves which are nodes of the standing wave. Consequently, we determine that the major contributions to the TPTE surface charge oscillation must be spherical harmonics $Y_{l}^{m}$ where l±m is even and l>0 .

iii) the transverse-propagating, axial-electric (TPAE) mode (Figure 7A right): This mode is excited when the electric field is polarized parallel to the rotational symmetry axis of the pJP. The surface charge density distribution exhibits a specific constraint due to coupling between the inner and outer surfaces of the gold cap. Specifically, at the poles, the surface charge density must maintain the same sign on both the inside and outside surfaces; otherwise, charge would flow through the volume, constituting a volume plasmon that requires significantly higher excitation frequencies. This constraint necessitates the presence of a nodal curve on each hemisphere that encircles the pole without intersecting the rim. Spherical harmonics $Y_{l}^{m}$ with |m|=l−2 satisfy this condition, and we therefore identify these as the fundamental representation of the TPAE mode.

From these constraints on the LSP modes, it follows that a surface plasmon resonance with *k*_SP_ = 1 · *R*_JP_^–1^ exists only in the TPTE and APTE modes, but not in the TPAE mode, where the minimum resonance wavenumber is 2 · *R*_JP_^–1^. This agrees with the analyses by King *et al.*,^34^ finding the scattering peak associated with the axial electric AE mode at a significantly shorter wavelength than that of the transverse electric (TE) modes.

To quantitatively analyze the modal composition, we extracted the electric field values distributed across the cap surface from numerical simulations and performed decomposition into spherical harmonics. Our analysis reveals that harmonics with *m* = ± 1 constitute the dominant contribution to the total field (Figure 7C). Quantitatively, all other harmonic components collectively contributed less than 1% to the total scattering power in the majority of cases, and never exceeded 3% across all examined configurations. These findings provide strong validation for our theoretical model of the APTE mode.

Each distinct peak observed in the scattering spectra corresponds to a localized surface plasmon resonance at the respective excitation frequency. By analyzing the spherical harmonic decomposition, we assigned a characteristic wavenumber $k=l\cdot {R_{\mathrm{JP}}}^{-1}$ to each spectral peak, where l represents the principal quantum number of the dominant harmonic $Y_{l}^{m}$ . The relationship between LSP wavenumber and excitation frequency, as demonstrated in Figure 7B, exhibits excellent agreement with the established plasmonic dispersion relation for interfacial systems^1,2,55^

$$\left|k_{\mathrm{SP}}\right|=\frac{\omega }{c}\sqrt{\frac{\epsilon_{1}\cdot \epsilon_{2}}{\epsilon_{1}+\epsilon_{2}}}$$

which is valid for planar interfaces between materials with permittivities ϵ_1_ and ϵ_2_ and used as an approximation here for the curved interface.

These multimodal excitations match our models for the transverse-propagating modes and explain why peaks in the side-on spectrum are spaced more closely and are less prominent. The degeneracy of the APTE mode explains the difference in magnitude between the transverse-illuminated and axially illuminated scattering cross sections, the latter being approximately twice as large. This, again, matches the observations of first-order resonances by King *et al.* ^34^

In the frequency range and size-parameter regime investigated here, the scattering response is dominated by electric surface charge oscillations associated with localized surface plasmons, and magnetic multipole contributions can therefore be neglected. Recent formulations of multipole decompositions in plasmonic systems provide a comprehensive electromagnetic classification of scattering responses;^58^ the present work instead adopts a system-specific, electric-only description tailored to surface-plasmon modes on asymmetric, yet topologically spherical interfaces.

We note that in scattering theory, the so-called Kerker effect can arise from interference between electric and magnetic multipoles, constituting suppressed backward or forward scattering under specific amplitude and phase relationships between the dipolar components. In our system, genuine magnetic dipole responses are negligible due to the low refractive index of the dielectric core and the weak magnetic response of noble-metal constituents at optical frequencies.^59^ The scattering spectrum is dominated by higher-order electric multipoles with minimal contributions from dipolar terms (l=1) . Consequently, interference effects associated with Kerker-type directionality are not expected to play a significant role in the observed scattering behavior.

Surface roughness from thermal evaporation introduces scattering centers that limit surface plasmon propagation. When the scattering length becomes comparable to the mode wavelength, coherent standing-wave formation is disrupted, leading to broadening and intensity reduction. We suppose that modes with circumferential wavelengths λ_circ_ > *L*_scat_ experience significant damping, providing a physical explanation for the observed spectral intensity drop-off at large wavelengths that is absent in our smooth-surface simulations.

## Conclusion and Outlook

In this work, we established Fourier Plane Tomographic Spectroscopy as a powerful analytical technique for characterizing orientation-dependent optical properties of anisotropic plasmonic particles. This methodology, integrating dark-field microscopy with angle-resolved spectroscopy, enables simultaneous mapping of wavelength-dependent scattering across multiple illumination and detection angles. Our experimental measurements demonstrate quantitative agreement with finite-element simulations, validating both our measurement approach and theoretical framework.

Our spectroscopic analysis reveals that the structural asymmetry of pJPs induces distinct splitting of surface plasmon modes, resulting in unique spectral signatures that directly correlate with particle orientation. We observed characteristic features—including a distinct shoulder at 550 nm and multiple resonance peaks extending into the near-infrared—that clearly differentiate these asymmetric structures from isotropic gold spheres. Our mathematical framework based on decomposition of electric multipoles as spherical harmonics quantitatively explains these spectral features by establishing selection rules for surface plasmon modes on the curved metallic interface. This approach successfully identifies axial-propagating transverse-electric, transverse-propagating transverse-electric, and transverse-propagating axial-electric modes that dominate the scattering response.

The mode-specific angular distribution patterns we observed offer new insights into the complex interactions between polarized light and asymmetric plasmonic structures. By quantitatively correlating spectral features with specific surface plasmon modes, we have established a foundation for determining pJP orientation from scattering spectra, which could significantly enhance their utility in active matter research and self-propelled particle tracking. Additionally, our comprehensive characterization of orientation-dependent plasmonic responses provides critical design parameters for engineering optical manipulation strategies and sensing platforms based on these asymmetric structures.

The analytical approach using spherical harmonic decomposition developed here provides a robust framework for investigating complex light–matter interactions in asymmetric plasmonic nanostructures with applications ranging from nanophotonics to biomolecular sensing. Future refinements could include real-time spectral analysis for dynamic orientation tracking and the extension of our mathematical framework to complex geometries beyond hemispherical caps. The quantitative relationships we established between structural asymmetry and plasmonic mode splitting offer valuable design principles for engineering orientation-sensitive plasmonic responses in next-generation metamaterials and active colloidal systems.

## Methods

### Sample Preparation

The particles under investigation were pJPs consisting of spherical polystyrene (PS) cores of 1 μm diameter hemispherically coated with gold. The gold coating was deposited to a thickness of 50 nm with an intermediate 5 nm chromium adhesion layer. Samples were prepared by first depositing a volume of 30 μL of the pJP suspension onto a glass coverslip. After allowing 10 min for sedimentation, the solvent was removed via nitrogen gas flow, resulting in pJPs immobilized on the coverslip surface. A second coverslip was placed on top with 1.5 μL of Immersol 518F immersion oil (Carl Zeiss Jena GmbH) between the two coverslips. This sandwich configuration provided a homogeneous refractive index environment around the particles (see Figure 1D).

Samples for the validation measurements were prepared in the same fashion, using AuNPs in place of the pJPs. The 65 nm AuNPs were purchased from NanoPartz Inc. (Product No. A11-65-CIT-DIH-1-25). The 250 nm AuNPs were obtained from Aldrich (Catalog No. 742074-25 ML). Both were used as received without further modification.

### Experimental Setup

The optical setup depicted in Figure 1 was constructed around an Olympus IX71 microscope platform. The numerical aperture of the dark-field condenser (Olympus U-DCW) ranged from 1.2 to 1.4, confining illumination to within 52.6° and 68.0° with respect to the optical axis. Aperture B1, constraining the illumination angle in-plane, was custom-manufactured with a slit width of 1 mm. Scattered light was collected using an Olympus UPlanFL N 100× objective with adjustable back aperture from 0.6 to 1.3 numerical aperture. All other lenses in the optical path were achromatic doublets with transmission spectral ranges from 400 to 1100 nm (Thorlabs AC-series). The spatial filter (B4) employed a pinhole with 0.9 mm diameter. The spectrograph consisted of a blazed transmission grating (Thorlabs GTI25-03A) mounted in front of an sCMOS camera sensor (PCO Edge 4.2), positioned behind an adjustable slit (Thorlabs VA100).

Switching between Fourier plane and real space imaging was facilitated by translating the lens L2 between its two calibrated positions along the optical axis. While real-space imaging was used to select individual particles and acquire scattering spectra without angular resolution, back focal plane (BFP) imaging provided spectrally resolved Fourier space scattering distributions. To acquire a 3D, angularly and spectrally resolved data set, the slit B5 was translated across the BFP image, enabling sequential capture of vertical lines of spectrally dispersed scattering information. This was repeated systematically, for multiple fixed orientations of aperture B1 to obtain complete multidimensional scattering maps.

For angular and spectral calibration, the objective’s back aperture (B3) was fully opened to its maximum numerical aperture of 1.3, allowing direct transmission of light from the dark-field illumination pathway into the imaging system. The image of the illumination pattern in the BFP was used to calculate the transformation from pixel coordinates to steric scattering angles. Meanwhile, the spectrally dispersed illumination pattern was used to determine the spectral response function of the setup, which is presented in Figure 1C. A detailed procedure for the acquisition and evaluation of calibration measurements is available as Supporting Information (Section S1). An optional long-pass filter could be used to red-shift the point of overlap between the first and second interference orders. Measurements with and without the filter were merged into spectroscopic datasets covering the full spectral range of the setup.

### Finite Element Simulations

Numerical simulations of the light–matter interaction of an individual pJP were performed using COMSOL Multiphysics 6.1. The pJP’s PS core was modeled as a sphere with diameter 1 μm and refractive index 1.58. Its cap was represented by a half-ellipsoidal shell around the core, with a thickness of 50 nm at the apex and 10 nm at the rim. For the refractive index of gold, we used the values reported by Johnson and Christy.^54^ This assembly was surrounded by an ambient medium with a constant refractive index of 1.51.

The simulations yielded numerical solutions for the scattered light field, given an incident plane wave. From the scattered field, optical cross sections, far-field scattering intensity distributions, and point-wise solutions for the electric field on the particle surface were calculated.

## Supplementary Material



## Notes

Detailed description of reference data acquisition procedure, discussion of signal-to-noise, additional notes on individual pJP dark-field spectra, quantitative analysis of agreement between measured and simulated angular scattering distributions, azimuthal intensity profiles, discussion of extinction spectra (PDF)
