# Supplementary material for: Fourier Plane Tomographic Spectroscopy Reveals Orientation-Dependent Multipolar Plasmon Modes in Micrometer-Scale Janus Particles
Source: ACS Nano. 2026 Mar 23;20(13):10696–706. doi: 10.1021/acsnano.6c01771 (PMC13063818; doi:10.1021/acsnano.6c01771)
Supplement: Supplementary file 1 [file nn6c01771_si_001.pdf]

Fourier Plane Tomographic Spectroscopy Reveals Orientation-Dependent Multipolar Plasmon Modes in Micrometer-Scale Janus Particles

Supporting Information

Felix H. Patzschke[1] and Frank Cichos[1*]
[1] Molecular Nanophotonics Group, Peter Debye Institute for Soft Matter Physics, Leipzig University, 04103 Leipzig, Germany
[*] corresponding author: cichos@physik.uni-leipzig.de

Contents

S1 Calibration and Measurement Procedure I

S2 Signal-to-noise considerations in angle-resolved scattering measurements III

S3 Individual pJP Dark-Field Spectra V

S4 Discrepancies between Experimental and Simulated Angular Distributions VI

S5 Azimuthal Profiles of the Scattering Intensity Distribution VII

S6 Extinction Spectra VIII

S1 Calibration and Measurement Procedure

Before each particle measurement, calibration data were recorded, from which the appropriate coordinate transformations and correction functions for the alignment and efficiency of the optical setup were determined. In this section, we describe, in detail, the sequence of changes to the optical configuration, image acquisitions and their places in the data evaluation pipeline.

S1.1 Spectral Dispersion Calibration

To facilitate calibration of the spectrograph sub-assembly, a low-power laser beam ($\lambda = 532\,\mathrm{nm}$) was directed into the objective using a beam splitter in the imaging path. With the space between the sample and the dark-field condenser not yet filled with immersion oil and due to the high numerical aperture ($\mathrm{NA} = 1.3$) of the fully opened back aperture B3 of the objective, part of the beam was reflected off the glass-air interface and subsequently directed back into the imaging path. With the lens L2 in the real-space imaging position and through the fully opened slit B5 at the front of the spectrograph, the laser beam was imaged into a spot at zeroth-, first- and second-order spots on the camera sensor behind the diffraction grating. The displacement between these spots was used to infer the distance between grating and sensor, allowing us to determine the wavelengths corresponding to each pixel in subsequently acquired images:

$$\Delta x = d \cdot \tan\left(\arcsin\left(\frac{m\lambda}{g}\right)\right) = \frac{dm\lambda}{\sqrt{g^2 - m^2\lambda^2}},$$

where Δx is the displacement between spots, d the distance between grating and sensor, g the line distance of the diffraction grating ($g = 3.33\,\mu\mathrm{m}$ in our case) and m the interference order. Displacements for varied lateral positions of the laser spot were compared to confirm precise alignment through the absence of distortions.

S1.2 Fourier Plane Calibration

Next, the laser was disengaged, the beam splitter removed and the illumination path connected to the remainder of the optical setup using a drop of immersion oil. With aperture B1 not yet installed, a reference image was recorded. This resulted of an image of the dark-field illumination ring, from which we determined the region of the image sensor, onto which the back focal plane would be imaged in the zeroth order.

Based on the range of the NA, restricted by the minimum NA of the dark-field condenser (1.2) and the maximum NA of the objective (1.3), this image also allowed us to construct the transformation from image coordinates to an angular coordinate w.r.t. the optical axis: Using the Abbe sine condition for the illumination ring,

$$\sin\left(\langle\theta\rangle_{\mathrm{ring}}\right) = \frac{\langle\mathrm{NA}\rangle_{\mathrm{ring}}}{n} \quad \text{with} \quad \mathrm{NA} \equiv \frac{r}{f},$$

where $n = 1.512$ is the refractive index, $\langle\theta\rangle_{\mathrm{ring}}$ is half the opening angle of the illumination cone and r is the distance of a point in the Fourier plane from the optical axis, we can determine the effective focal distance f of the entire imaging assembly. By re-substituting into the Abbe sine condition for an arbitrary point in the BFP, we obtain

$$\sin\theta = \frac{\langle\mathrm{NA}\rangle_{\mathrm{ring}}}{n \cdot r_{\mathrm{ring}}} \cdot r\,,$$

where r is the distance from a point to the centre of the ring in pixels and θ is the corresponding angle between the direction of light propagation and the optical axis. The centre of the ring corresponds to the optical axis.

This coordinate transformation was used not only to determine a meaningful angular domain, but also for the calculation of the apodization factor, which measured intensities had to be corrected for. [Ref. 52] The ratio between mapped solid angle element and camera sensor area element depended on the angular deviation from the optical axis:

$$\mathrm{d}\Omega = \frac{\mathrm{d}A}{n^2 f^2 \cos\theta},$$

where $\mathrm{d}A$ and $\mathrm{d}\Omega$ are infinitesimal area and solid angle elements, respectively. Measured intensities $(I = \mathrm{d}P/\mathrm{d}A)$ were corrected for this ratio to recover scattered light intensity with respect to scattering angle, $\mathrm{d}P/\mathrm{d}\Omega$.

S1.3 Spectral Efficiency Calibration

Next, the aperture B5 was closed to a narrow slit and another image was recorded. This resulted in only a narrow strip of the illumination pattern being imaged into the spectrograph. From this image, we extracted the spectral response function of the entire optical assembly, which is shown in Figure 1C in the main text. Spectrally dispersed intensity maps were divided by this response function.

S1.4 Target Selection

Then, the lens L2 was moved to the real-space-imaging position, the objective's back aperture was narrowed to completely block the illumination from passing through and the slit B5 was opened slightly to facilitate the selection of a particle. This selection was facilitated by an xyz Piezo stage, precisely translating the sample to line up a particle with the optical axis.

S1.5 Establishment and Measurement of the Selective Illumination Profile

With a particle selected, the restricted illumination would be established by installing aperture B1. The objective's back aperture B3 and the slit B5 would be fully opened and the lens L2 moved to the Fourier-plane-imaging position again to obtain an image of the selective illumination profile. From this, we extracted the in-plane illumination angle. The out-of-plane illumination angle was already known as it is entirely defined by the NA of the dark-field condenser:

$$\sin\theta_{\text{illumination}} = \frac{\langle \text{NA} \rangle_{\text{condenser}}}{n} \approx 59.7^\circ$$

Using these two values, the required rotation of the coordinate frame from angles of deviation from the optical axis to scattering angles (w.r.t. incident light) was determined.

S1.6 Final Configuration for Fourier Tomographic Plane Spectroscopy

Finally, the objective's back aperture B3 was narrowed to block the illumination again. The slit B5 would be narrowed to a width of 20 µm and slowly translated over the BFP image while recording a video.

In the analysis, the horizontal position of the slit would be extracted per-frame by thresholding over the pre-determined region of the zeroth order image and the horizontally spectrally dispersed intensity profiles would be accumulated into a 3D array with axes corresponding to vertical pixel coordinate, horizontal pixel coordinate in the zeroth order and wavelength. Then the previously determined coordinate transformations were applied.

S2 Signal-to-noise considerations in angle-resolved scattering measurements

In angle-resolved scattering spectroscopy, the detected signal at a given wavelength and scattering angle corresponds to the number of photo-electrons accumulated within a specific angular bin on the camera sensor. The signal-to-noise ratio (SNR) in each bin is therefore determined by photon statistics, detector noise, and experimental stability.

S2.1 Photon statistics and angular binning

For an incident photon flux $\Phi_0(\lambda)$, the mean number of photons scattered by a particle during an exposure time T is given by

$$\langle N_{\text{sca}} \rangle \propto \Phi_0(\lambda) \cdot \sigma_{\text{sca}} \cdot T ,$$

where σ_{sca} is the total scattering cross-section. In angle-resolved measurements, these photons are redistributed over solid angle according to the angular scattering function $f(\Omega)$. The mean number of detected photons in an angular bin $\Delta\Omega$ is therefore

$$\langle N_{\text{bin}} \rangle \propto \Phi_0(\lambda) \cdot \sigma_{\text{sca}} \cdot \frac{f(\Omega)\,\Delta\Omega}{4\pi} \cdot \eta \cdot T ,$$

where η is the camera quantum efficiency of the sensor. Assuming Poisson photon statistics, the corresponding shot noise is $\sqrt{\langle N_{\text{bin}} \rangle}$, yielding an SNR that scales as $\sqrt{\langle N_{\text{bin}} \rangle}$. Increasing angular resolution (smaller $\Delta\Omega$) therefore reduces the SNR per bin linearly in $\Delta\Omega$ and cannot be compensated without increasing the total number of scattered photons.

S2.2 Dependence on particle size

For particles well below the wavelength (Rayleigh regime), the scattering cross-section scales as $\sigma_{\text{sca}} \propto r^6$, resulting in rapidly decreasing photon counts per angular bin for smaller particles. In this regime, angular scattering patterns are predominantly dipolar and weakly wavelength dependent, such that angle-resolved measurements mainly probe overall intensity rather than angular redistribution, hence the applicability of small particles to the validation of angle-independent spectroscopic performance. For larger particles approaching the Mie regime $x \approx 1$, σ_{sca} increases by orders of magnitude and the angular distribution exhibits wavelength-dependent multipolar structure. This simultaneously increases the photon budget per angular bin and provides non-trivial angular features that allow a meaningful validation of the angular resolution of the measurement.

S2.3 Exposure time, detector dynamic range, and averaging

In principle, photon-shot-noise-limited SNR improves with exposure time T. In practice, the exposure per frame is chosen to utilize the full dynamic range of the detector without saturating the brightest angular–spectral bins. The limitation then arises in the weakest bins, where the signal may be comparable to or smaller than the background level arising from residual stray light, dark current, and other sources of background leakage.

Averaging multiple shorter exposures improves photon shot noise statistics but does not recover weak features buried in background: because background leakage accumulates proportionally with integration time, averaging N frames increases both signal and background by a factor of N, leaving the signal-to-background ratio unchanged. Combined with slow intensity drifts of the illumination source, this places a fundamental limit on the achievable SNR in the weakest angular–spectral bins.

For the sCMOS detector used here (full-well capacity $\sim 3 \times 10^4\,\text{e}^-/\text{px}$, read noise $\sigma_{\text{read}} \sim 1.5\,\text{e}^-/\text{px}$ RMS, dark current $I_{\text{dark}} \sim 1\,\text{e}^-/\text{px/s}$), the total noise in a given angular–spectral bin is

$$\sigma_{\text{total}} = \sqrt{N_{\text{det}} + N_{\text{bg}} + \sigma_{\text{read}}^2 + I_{\text{dark}} \cdot T} ,$$

where N_{det} is the number of detected signal photoelectrons and N_{bg} is the background contribution from stray light. At a typical exposure time of $T = 2\,s$, adopting a conservative threshold of SNR $\geq$ 3 for resolving angular features requires a minimum of $N_{det} \approx$ 10–15 photoelectrons per bin above background. This photon budget sets the practical detection limit for weak scattering features.

S2.4 Implications for particle size selection

Using Mie-theory scattering cross-sections for spherical gold nanoparticles, we estimate that particles with diameters below approximately 100–150 nm would produce photon counts per angular bin that fall below the above SNR threshold over substantial portions of the wavelength and angular range, particularly away from the plasmon resonance where the scattering cross-section decreases rapidly. This limitation arises from the large intensity contrast between the brightest and weakest angular–spectral bins, which exceeds the usable dynamic range of the detector for a single unsaturated exposure.

In contrast, the 250 nm AuNPs used for validation provide photon counts that exceed the SNR threshold across the full angular and spectral range, while simultaneously exhibiting wavelength-dependent multipolar scattering that produces non-trivial angular structure. The strong agreement between experiment and theory (Fig. 2B in the main text) therefore confirms that particle size constraints are comfortably satisfied and that the technique operates well within its practical limits for the particle sizes investigated in this work.

S3 Individual pJP Dark-Field Spectra

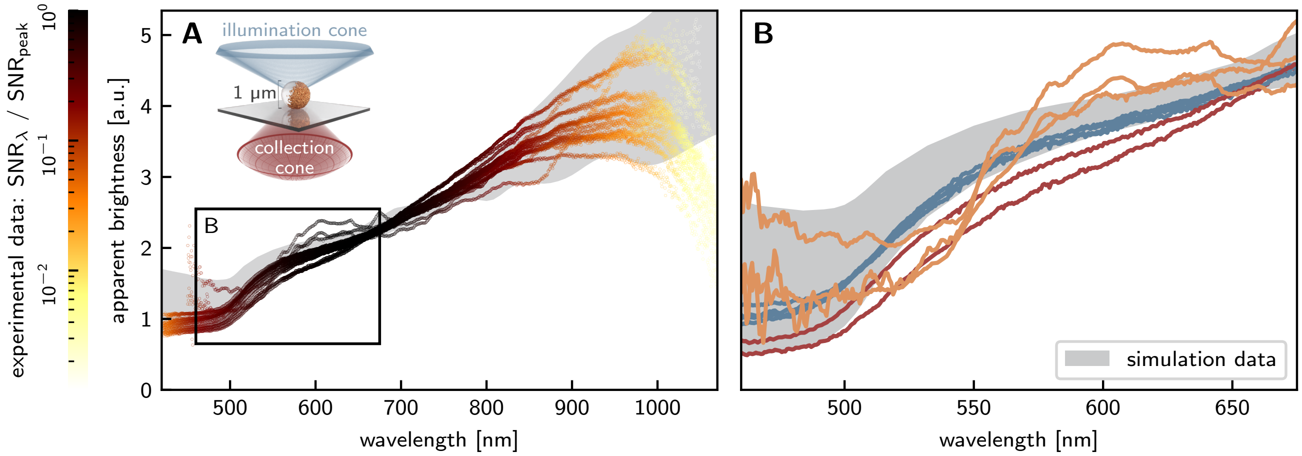

Figure S1: A: Measured scattering spectra of 1 µm pJPs under standard dark-field illumination. Light is incident from a range of directions simulateneously, as depicted in the sketch. The shaded area depicts the range of simulated measurements. **B:** Magnified view of the spectral range of the shoulder feature. A selection of measured spectra (lines, all colours) is overlaid on the range of simulated spectra (shaded area).

In Figure S1A, we present the individual measured spectra of all analyzed 1 µm pJPs under standard dark-field illumination.

The dark-field spectra exhibit some variation, which was expected due to the out-of-plane orientation of the JPs and the exact NA setting of the objective being uncertain. Accordingly, we generated synthetic measurements for a range of combinations of these parameters ($0.8 \leq \mathrm{NA} \leq 1.2$ and any out-of-plane orientation for the pJP). All spectra were L^2-normalized to the spectral range from 450 nm to 850 nm before comparison.

An expanded view with a representative selection of measured spectra is presented in Figure S1B. Here, it is apparent that experimental data don't match the simulation results perfectly: Some spectra exhibit the shoulder weakly (blue curves), systematically erring closer to the lower bound of the range of simulated data, some very weakly (red curves), deviating below all synthetic spectra. Others exhibit the shoulder more strongly (yellow curves) but with a significant red-shift.

S4 Discrepancies between Experimental and Simulated Angular Distributions

Agreement between experimental and simulated angular profiles was assessed using normalized root-mean-square deviation (NRMSD). Measured datasets were aligned to the simulated scattering intensities only through scaling by constant normalization factors. Simulated profiles were linearly interpolated at points corresponding to the measurements. NRMSDs were calculated as

$$\frac{\sqrt{\frac{1}{N}\sum_{i=1}^{N}\left(I_{\text{exp}}(\theta_i) - I_{\text{sim}}(\theta_i)\right)^2}}{\max\ I_{\text{sim}}(\theta_i)}$$

for each illumination mode and fixed wavelength sample.

As we demonstrate in Figure S2A, NRMSD values ranged from 1.06% to 9.45% across the measured spectral range and all illumination configurations, indicating solid agreement. The primary discrepancies occur at scattering angles where intensity, and thus signal-to-noise is extraordinarily low, as can be seen in Figure S2B-D. Such local minima may, under variation of any parameter (particularly wavelength or orientation), appear quite suddenly, as they are caused by destructive interference of the scattered light field. Hence, with parameters only slightly off from the ground truth, simulations may fail to reproduce or erroneously predict such features.

Moreover, the Au caps of real pJPs do not have perfectly smooth surfaces and sharp boundaries, as can be seen in the SEM image in Figure S2E. Surface and boundary imperfections constitute additional scattering centres which the simulation does not take into account. We expect these to add an approximately isotropic noise component to the scattered field, further obscuring the minima.

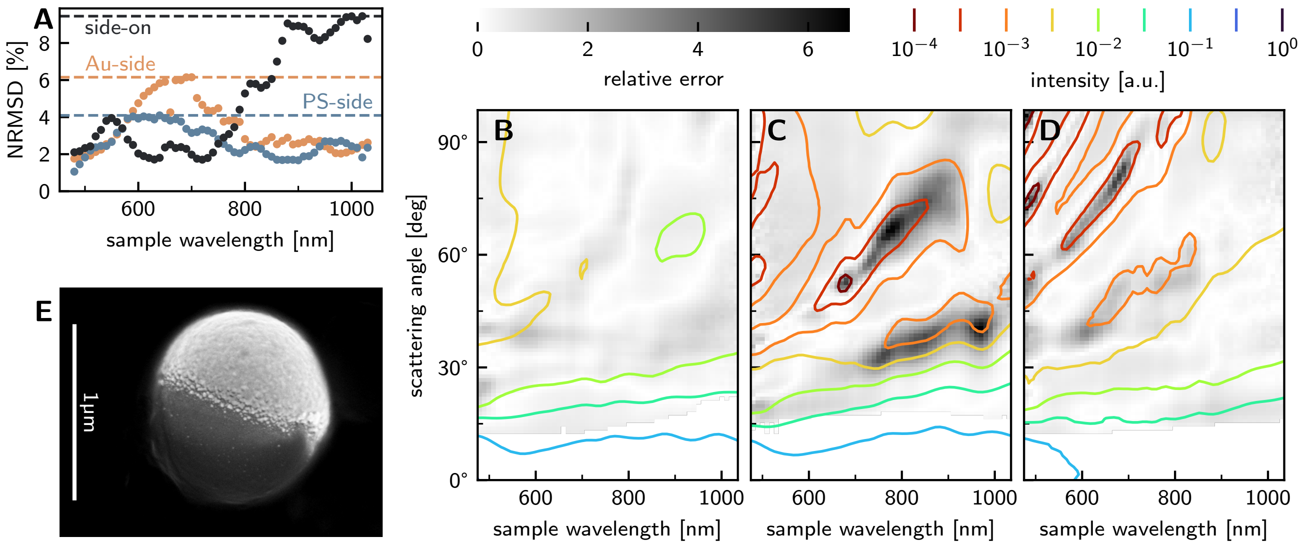

Figure S2: A: NRMSD between measured and simulated angular profiles. The colours indicate the illumination mode corresponding to each dataset. **B-D:** Relative error of measured intensities w.r.t simulation results. The overlaid contours represent the value of the simulated scattering intensity. Spikes in the relative error coincide with minima in the simulation results. **B:** Au-side illumination, **C:** PS-side illumination, **D:** side-on illumination. **E:** SEM image of a pJP. The Au cap has no solid boundary; instead, the rim comprises disconnected Au droplets.

S5 Azimuthal Profiles of the Scattering Intensity Distribution

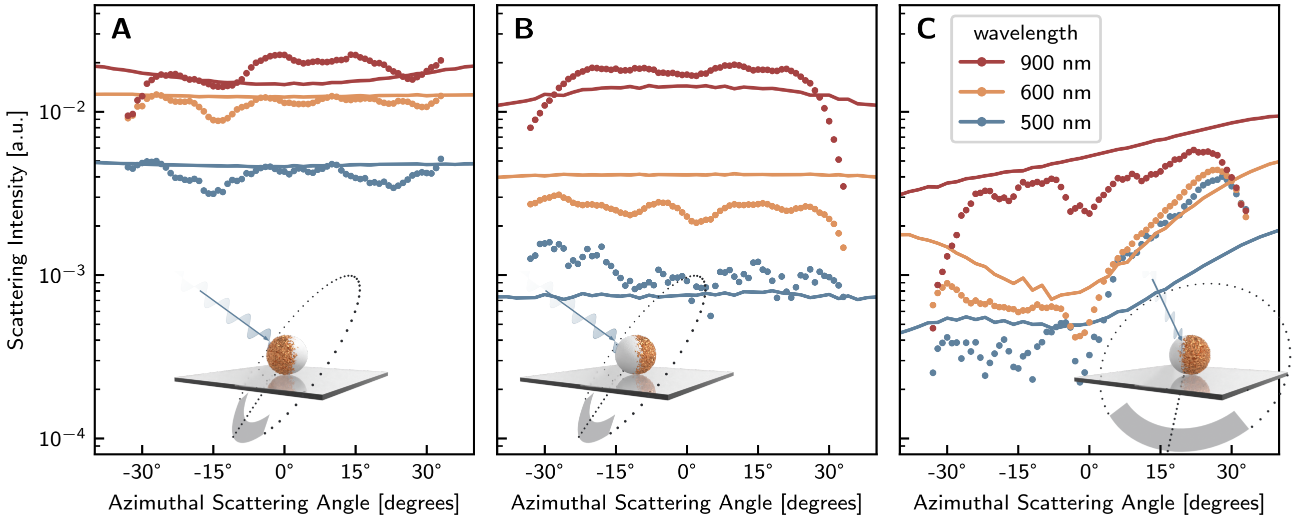

Figure S3: Intensity of scattered light at a selection of wavelengths versus azimuthal coordinate of the scattering angle. The polar coordinate is held fixed at 90°. Points indicate measured intensities; lines show simulated profiles. The orientations are illustrated in the respective insets, the grey shaded areas corresponding to the azimuthal range. **A:** illumination from the Au side. **B:** illumination from the PS side. **C:** side-on illumination. Here, positive azimuth corresponds to the direction of the Au side, negative azimuth to the PS side.

The intensity of scattered light also varied w.r.t. the azimuthal coordinate of the scattering angle. Measured and simulated profiles are presented in Figure S3, showing solid qualitative agreement: Under illumination from either the Au side (Fig. S3A) or the PS side (Fig. S3B), the azimuthal distribution are evenly symmetric, as expected due to the system's symmetry about the plane of the optical and illumination axes. Moreover, the distributions are close to flat within the imaged range of scattering angles.

For shorter wavelengths, scattering intensities are lower under PS-side illumination than under Au-side illumination, whereas for longer wavelengths, they appear to assimilate. This trend is consistent with the data we present in Figures 3 and 6B of the main text.

Under side-on illumination (fig. S3C), the geometrical symmetry of the system is broken. Both experimental and simulated data show that scattered light is preferentially emitted in the direction of the Au cap, rather than the PS side of the pJP.

The drop-off that is seen in all measured azimuthal profiles for azimuths outside ±30° corresponds to the boundary of the collection cone of the objective.

S6 Extinction Spectra

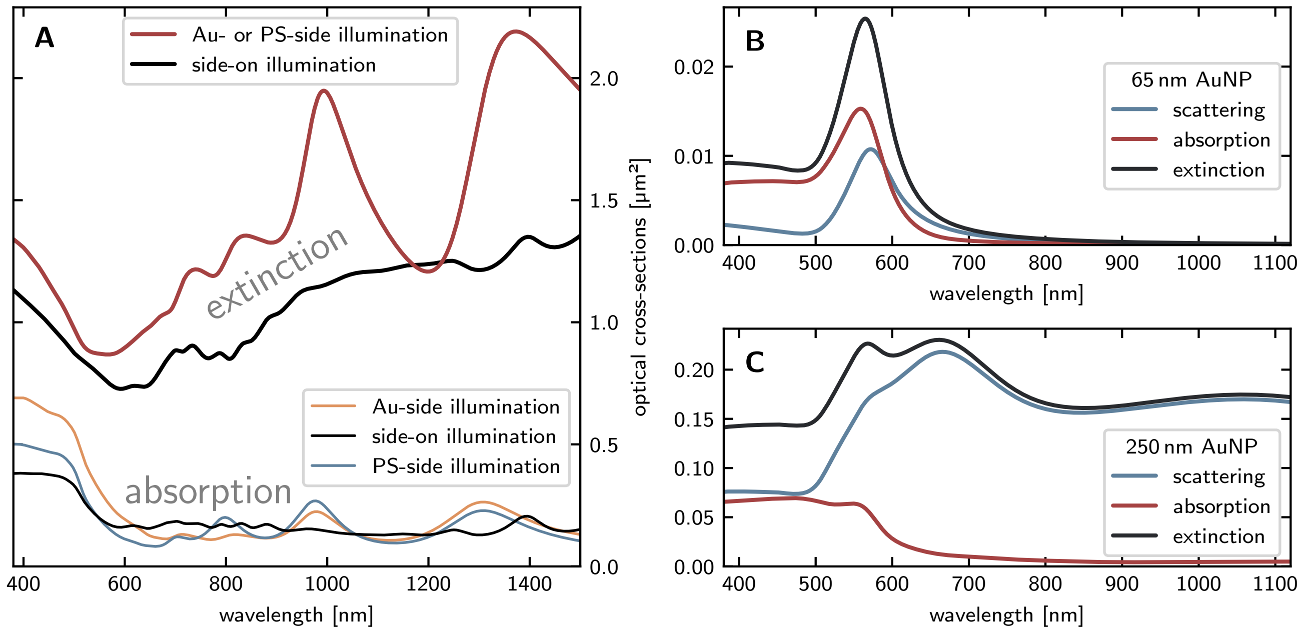

Figure S4: A: simulated absorption and extinction spectra of the pJP for relevant illumination modes. While the absorption spectra differ substantially between Au side (yellow) and PS side illumination (blue), the extinction spectra for either illumination mode (red) are virtually identical. The black lines correspond to side-on illumination for either interaction cross-section. **B–C:** Scattering, absorption and extinction spectra of solid AuNPs, 65 nm **(B)** and 250 nm **(C)** in diameter, respectively. Here, the optical cross-sections were calculated from Mie theory.

The numerical simulations readily yielded values for the orientation-dependent absorption cross-sections of the pJP, from which we calculated the extinction as well. Both are presented in Figure S4A. Absorption, scattering and extinction spectra of the AuNPs were calculated directly *via* Mie theory and are presented in Figures S4B and C.

For the 65 nm AuNPs, absorption and scattering contribute comparably to the extinction (see Fig. S4B). Meanwhile, for the 250 nm AuNPs (Fig. S4C) and the pJPs (Fig. S4A), extinction is dominated by scattering, owing to their larger size, with absorption contributing significantly only at shorter wavelengths, near the interband transition of gold. As such, the pJP's extinction spectra possess characteristic orientation-dependent peaks, similar to those of the scattering spectra discussed in the main text.

While, separately, the absorption and scattering cross-sections of the pJP differ substantially between Au-side and PS-side illumination (as discussed in the main text), the total extinction cross-sections are nearly identical for both orientations. We calculated a root mean square deviation of 1.31%, which we attribute to numerical errors. More generally, the extinction cross-section is invariant under reversal of the incident field's wave vector (in the reference frame of the pJP), due to the time-reversal symmetry of Maxwell's equations for linear, passive media.
